# Age and Sex Pattern of Cardiovascular Mortality, Hospitalisation and Associated Cost in India

**DOI:** 10.1371/journal.pone.0062134

**Published:** 2013-05-07

**Authors:** Akanksha Srivastava, Sanjay K. Mohanty

**Affiliations:** 1 International Institute for Population Sciences, Mumbai, India; 2 Department of Fertility Studies, International Institute for Population Sciences, Mumbai, India; Tehran University of Medical Sciences, Iran (Republic of Islamic)

## Abstract

**Context:**

Though the cardiovascular diseases are the leading cause of mortality in India, little is known about the human and economic loss attributed to the disease. The aim of this paper is to account the age and sex pattern of mortality, hospitalisation and the cost of hospitalisation for cardiovascular diseases in India.

**Data and Methods:**

Data for the present study has been drawn from multiple sources; 52^nd^ and 60^th^ rounds of the National Sample Survey, Special Survey of Death, 2001–03 and the Sample Registration System 2004–2010. Under the changing demographics and constant assumptions of mortality, hospitalisation and cost of hospitalisation, we have estimated the deaths, hospitalisation and cost of hospitalisation for cardiovascular diseases in India during 2004 to 2021. Descriptive analyses and multivariate techniques were used to understand the socio-economic differentials in cost of hospitalisation for cardiovascular diseases in India.

**Findings:**

In India, the cardiovascular diseases accounted for an estimated 1.4 million deaths in 2004 and it is likely to be 2.1 million in 2021. An estimated 6.7 million people were hospitalised for cardiovascular diseases in 2004, and projected to be 10.9 million by 2021. Unlike mortality, majority of the hospitalisation due to cardiovascular diseases will be in the prime working age group (25–59). The estimated cost of hospitalisation for cardiovascular diseases was 94/− billion rupees in 2004 and expected to be 152/− billion rupees by 2021, at 2004 prices. The cost of hospitalisation for cardiovascular diseases was significantly high in private health centres, high fertility states and among high socio-economic groups.

**Conclusion:**

The cardiovascular mortality and hospitalisation will be largely concentrated in the prime working age group and the cost of hospitalisation is expected to increase substantially in coming years. This calls for mobilising resources, increasing access to health insurance and devising strategies for the prevention, control and treatment of cardiovascular diseases in India.

## Introduction

Cardiovascular diseases (CVDs) have emerged as a major public health challenge globally. Recent estimates indicate a rising trend in CVD deaths from 17 million in 2002 to 23 million in 2030, with a distinct growth pattern in developed and developing countries [1]. While, there is a preponderance of CVD deaths in older ages, in developed countries, it is more pronounced among the younger population, in developing countries [2], [3]. The onset of cardiovascular diseases in developing countries is 10–15 years earlier than that of developed countries [4]. In recent decades, there has been growing research interest on disease burden and implications of CVDs in developing countries [5]–[16]. Numerous studies have also envisaged the adverse economic consequences of growing CVDs both at micro and macro level [17]–[23].

India, with a population of 1.2 billion, is experiencing an increase in longevity and income levels, reduction in fertility and increasing burden of CVD mortality and morbidity. Evidence from the special survey on cause of death (2001–2003) suggests that the CVDs are the leading cause of death in India; accounting for one-fifth of all deaths [24]. According to medical certification of cause of death (MCCD), the proportion of deaths due to diseases of the circulatory system (cardiovascular diseases) was 20.4% in 1990 and increased to 27.1% in 2004 [25], [26]. Besides high burden, the disease is increasingly affecting the working population. For instance, CVDs accounted for 24.8% of the total deaths in the age group 25–69 and 25.7% among 70 years and above [24]. The mean age of first myocardial infarction is 53 years among Indians; which is about 10 years earlier than their counterparts in developed countries [4]. While earlier studies have shown a positive gradient of CVDs with socio-economic status [27], [28], recent studies documented CVDs as the major cause of death among poor, less educated and in rural areas [29]–[32]. The CVDs not only account for a higher proportion of deaths, it is one of the leading causes of hospitalisation in the country [23], [33]. The private sector is the main provider of inpatient and outpatient services for CVDs [34], [35]. The median cost of treatment for CVDs in India varies from US $773 in low income group to US $2917 in high income group (2007 prices) [18]. The macro estimates on loss in annual household income in India were in the range of Rs 144–158/− billion for heart disease and Rs 199/− billion for hypertension in 2004 [23]. The WHO estimated a loss of US $9 billion in India’s national income from premature deaths due to heart disease, stroke and diabetes in 2005 and US $235 billion in 2015 [1]. The number of years of life lost due to coronary heart disease deaths in India, below 60 years, is estimated to be 17.9 million in 2030, higher than that of China, USA and Russia combined [35]. The burden of CVDs is likely to grow cutting across the socio economic groups [36], [37].

The aim of this paper is to project the deaths, hospitalisation and cost of hospitalisation for cardiovascular diseases in India. The exercise was carried out for 2004 (base year) and projected for 2010, 2016 and 2021. The paper has been conceptualised with the following rationale. First, though the CVDs are the leading cause of death in India, little is known about the changing age and sex pattern of mortality and hospitalisation due to CVDs. An attempt to estimate the current and future scenario of mortality and hospitalisation by age and sex will be helpful for evidence based policies in prevention and control of CVDs. Second, evidences suggest that the treatment of CVDs is expensive, need specialised medical care, mostly provided by the private health centres and often tend to be catastrophic [18], [34], [35]. On the other-hand, the public health centres in India, that largely caters to the poor and disadvantageous, are not equipped to provide the curative services for CVDs. Third, though a number of studies have analysed the risk factors of CVDs, few studies have examined the socio-economic differentials in cost of hospitalisation for CVDs [33]. Fourth, the high level expert group (HLEG) has recommended increase in public spending on health from 1% of GDP in 2010 to 3% by 2020 [38]. Thus, increasing the budgetary allocation for cardiovascular diseases is of prime significance that would effectively save human lives and reduce the economic burden to the households.

## Materials and Methods

The data were analysed anonymously, using publicly available secondary data, therefore no ethics statement is required for this work.

### Data

The study utilises the unit and aggregate data from multiple sources as no single source provides comprehensive information on mortality and hospitalisation for CVDs in India. The unit data were drawn from the 52^nd^ and 60^th^ rounds of the National Sample Survey (NSS) conducted during July 1995-June 1996 and January-June 2004 respectively, by the National Sample Survey Organization (NSSO), Government of India. The two rounds of the NSS are the only data bases that provide comprehensive and comparable information on morbidity pattern and health care utilisation among the general population in the country. The schedule 25 of 52^nd^ and 60^th^ rounds on morbidity and health care broadly covered type of aliment, duration of ailment, health care utilisation and expenditure incurred for each member of the sampled household for both hospitalisation and outpatient services. The hospitalisation includes those who sought treatment as inpatient in a reference period of 365 days, while, the outpatient services had a reference period of 15 days. Details of the sample design, survey findings and the instruments are available in the national report [33], [39]. The aggregate data were obtained from the Special Survey of Deaths (SSD), 2001–03, the Sample Registration System (SRS) 2004–2010 and the population total of Census of India 2001 and 2011. The SSD 2001–03 is a collaborative effort of Office of Registrar General of India (ORGI) and Centre for Global Health Research (CGHR), Canada. The survey is based on the advanced form of verbal autopsy, which covered all the deaths under SRS during 2001 to 2003. The SSD is a benchmark study that provides comprehensive and reliable estimates on causes of death in India. The rationale, design and the findings of the study are available elsewhere [24], [40], [41].

The age and sex distribution of the population for 2004, 2010, 2016 and 2021 is one of the key inputs in estimating deaths, hospitalisation and cost of hospitalisation. We have used the age distribution and age specific death rates for 2004 and 2010 from the SRS [42], [43]. The total population for 2004 and 2010 (1^st^ March) was estimated using the inter-censal growth rate (2001–11) and combined with the age-sex distribution to obtain population by age and sex. The projected population for the year 2016 and 2021 was borrowed from the expert committee report on population projections for India and states [44]. We have preferred to use the population projection of the expert committee because of its wider acceptability. Also, the population projection of the expert committee for the year 2011 was close to the census count [44], [45]. The ASDR for 2016 and 2021 was borrowed from the revised United Nations Model Life Table (South Asian pattern) [46].

### Methods

The study derives macro estimates of mortality, hospitalisation and cost of hospitalisation by broad age group and sex in India. We have carried out the exercise for the year 2004, 2010, 2016 and 2021. The year 2004 is taken as base as most of the data pertain to the same period. Under the assumption of constancy in proportion of CVD deaths, hospitalisation rate, mean cost (2004 prices), and changing age and sex structure, we have projected the deaths, hospitalisation and cost of hospitalisation for 2010, 2016 and 2021.

To estimate total deaths due to CVDs, the following mathematical equation is used;
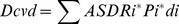
(1)where, D_cvd_ is the total number of deaths due to cardiovascular diseases.

ASDR_i_ is the age specific death rate in the i^th^ age group.

P_i_ is the population in the i^th^ age group.

d_i_ is the proportion of cardiovascular deaths to total deaths in the i^th^ age group.

To estimate total hospitalisation due to CVDs, the following mathematical equation is used;
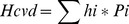
(2)


where, H_cvd_ is the total number of hospitalisation due to cardiovascular diseases.

h_i_ is the hospitalisation rate for cardiovascular diseases in the i^th^ age group.

The hospitalisation rate is defined as the total number of episodes of hospitalisation due to cardiovascular diseases per 1000 exposed population.

P_i_ is the population in the i^th^ age group

Similarly, the cost of hospitalisation due to CVDs was estimated using the following mathematical equation:
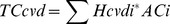
(3)


Where TC_cvd_ is the total cost of hospitalisation for cardiovascular diseases.

H_cvd i_ is the hospitalisation for cardiovascular diseases in i^th^ age group.

AC_i_ is the average cost of hospitalisation for cardiovascular diseases in the i^th^ age group.

Table 1 presents the key inputs and assumptions used in the projection of mortality, hospitalisation and cost of hospitalisation for CVDs in India. The key inputs are age and sex distribution of population, age specific death rate, proportion of CVD deaths, hospitalisation rate and cost of hospitalisation. With respect to mortality projections, while the age distribution of population and the ASDR are varied for the projected period, the proportion of CVD deaths is assumed to be constant at the base level. Similarly, the estimates of hospitalisation and associated cost were projected by varying the age distribution, and constancy in hospitalisation rate and cost at the base level. It may be mentioned that the population estimates are fairly accurate as we have estimated the population for 2004 and 2010 using inter-censal estimates and age-sex distribution of SRS. The projected population for 2016 and 2021 is borrowed from expert committee population projection. Similarly, the ASDR for 2004 and 2010 are observed [42], [43]. The proportion of CVD deaths for 2004 is obtained from the Special Survey of Deaths. The hospitalisation rate and the cost of hospitalisation for 2004 are derived from unit data of NSS, 2004. We have also provided the confidence interval for hospitalisation and cost of hospitalisation by broad age group (Table S1).

**Table 1:** Assumptions on projecting deaths, hospitalisation and cost of hospitalisation for cardiovascular diseases, India, 2004–21.

	2004 (Base year)	2010	2016	2021
**A- Inputs on estimating mortality due to CVDs**				
Proportion of deaths due to CVDs	Estimates from Special Survey of Deaths, 2001–03	Constant at 2004 level	Constant at 2004 level	Constant at 2004 level
Age specific death rate	Sample Registration System, 2004 (Observed)	Sample Registration System, 2010 (Observed)	UN model life table (based on Lee carter approach)	UN model life table (based on Lee carter approach)
Age distribution of population	SRS age distribution and intercensal population for 2004	SRS age distribution and inter censal population for 2010	Expert group population projection (based oncohort componentmethod)	Expert group population projection (based on cohort component method)
**B-Inputs on estimating hospitalisation due to CVDs**				
Hospitalisation rate	Computed from NSS 2004(Observed)	Constant at 2004 level	Constant at 2004 level	Constant at 2004 level
Age distribution of population	Same as mortality projection	Same as mortality projection	Same as mortality projection	Same as mortality projection
**C-Inputs on estimatin**g **cost of hospitalisation**				
Real cost of hospitalisation and hospitalisation rate-	Computed from NSS 2004(Observed)	Constant at 2004 level andprices	Constant at 2004 level and prices	Constant at 2004 level and prices
Age distribution of population	Same as mortality projection	Same as mortality projection	Same as mortality projection	Same as mortality projection

### Sensitivity Analysis

**Figure 1:**
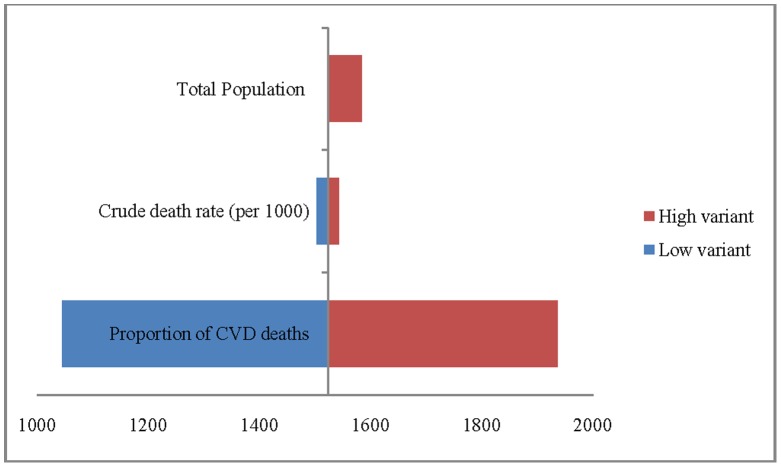
Sensitivity analysis for the estimates of CVD deaths by varying three parameters, India, 2004.

To examine the precision of the estimated CVD deaths, we have carried out the sensitivity analyses by varying three parameters; total population, crude death rate (CDR) and the proportion of CVD deaths to total deaths. In the absence of the confidence interval, the sensitivity analysis is used to examine the sensitivity of the estimates to changes in its inputs [47], [48]. For 2004, we observed that the estimated CVD deaths are less sensitive to population change and crude death rate (Figure 1). For example, the estimated number of deaths due to CVDs in India, in 2004, was 1.52 million and varied in the range of 1.50–1.54 million under low and high population variant respectively. Similarly, the estimated deaths by varying the CDR were in the range of 1.52–1.58 million. The estimated deaths were, however, more sensitive to the proportion of CVD deaths and varied in the range of 1.04–1.93 million for 2004. Based on these results, we have further carried out the sensitivity analyses for 2010, 2016 and 2021 by varying the proportion of CVD deaths only. We have used the estimated inter-censal population as standard, population projection of the expert committee as low variant and the UN population projection (high variant) as high [49]. We have used the observed CDR and 95% confidence interval as low and high variant [42], [43]. Similarly, the observed proportion of CVD deaths for Empowered Action Group States **(**the states of Bihar, Chhattisgarh, Jharkhand, Madhya Pradesh, Odisha, Rajasthan, Uttarakhand and Uttar Pradesh) and Assam was taken as low variant and all other states as high variant [24]. To provide the precision of estimates of CVD deaths by age and sex, we assume a uniform increase of 5% (medium variant) and 10% (high variant) of CVD deaths for 2010 across age groups, from 2004 level. The increase was 10% (medium variant) and 15% (high variant) for 2016 and 15% (medium variant) and 20% (high variant) for 2021.

In addition to the macro estimates, we have provided the mean cost of hospitalisation for CVDs by population subgroups. Descriptive statistics, bi-variate analyses and a log transformed ordinary least square (OLS) regression model are used to understand the socio-economic differentials and significant predictors of cost of hospitalisation for CVDs. The log transformed model is used as there were very few zero cases (less than 40) and the distribution of the dependent variable (cost of hospitalisation for CVDs) was skewed. Those who reported zero expenditure were excluded from the regression analyses. The dependent variable is in logarithm form (log of cost of hospitalisation for CVDs).

## Results

Results are presented in two sections. Section I presents the estimated number of deaths, hospitalisation and the cost of hospitalisation for cardiovascular diseases. Section II describes the socio-economic differentials in cost of hospitalisation for cardiovascular diseases.

### Section I

#### Estimated deaths due to cardiovascular diseases in India, 2004–21

**Figure 2:**
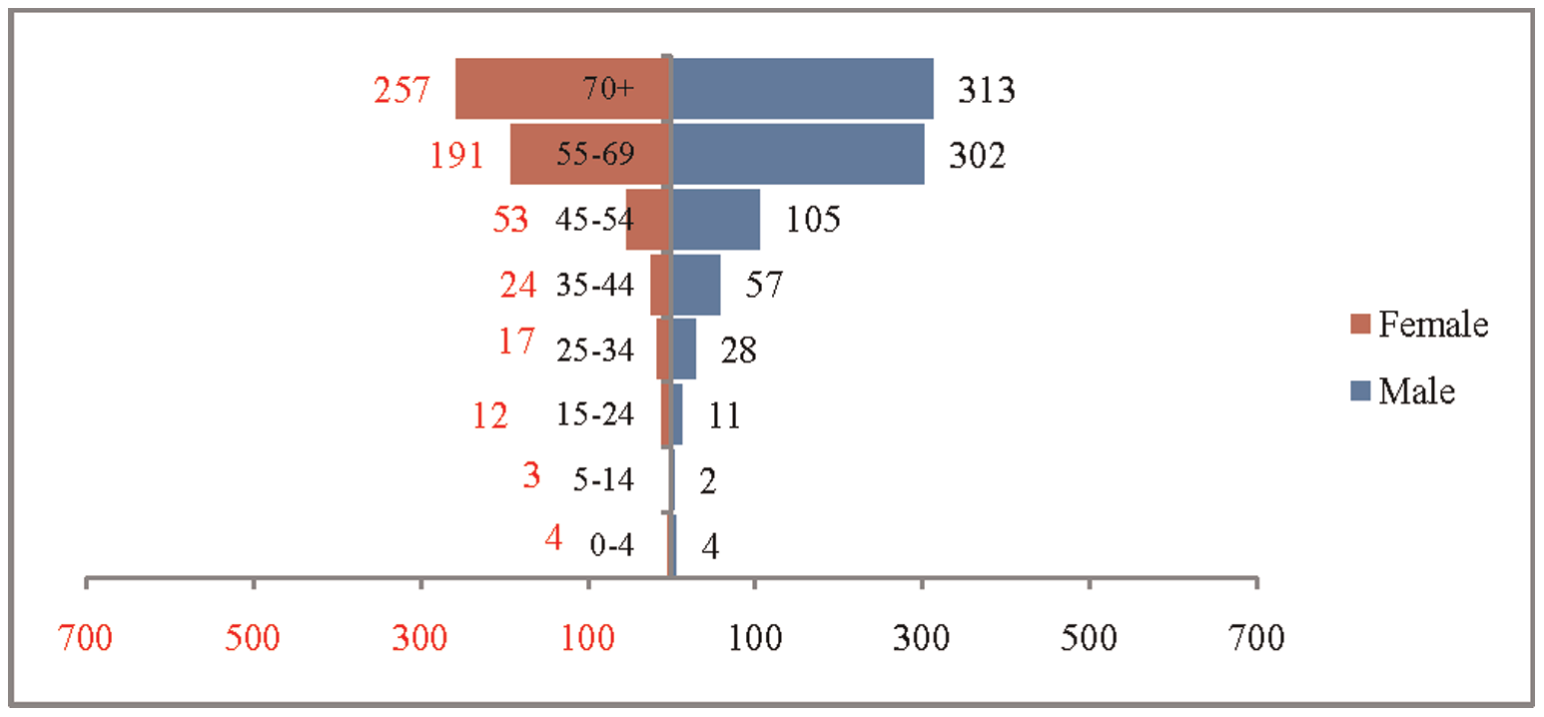
Estimated number of deaths due to CVDs (in 000’s) by age and sex, India, 2004.

**Figure 3:**
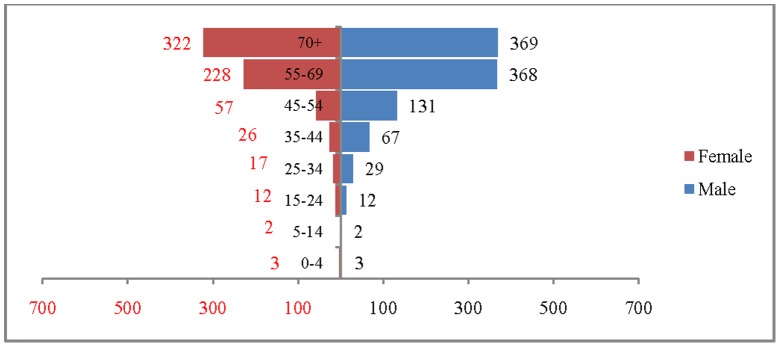
Estimated number of deaths due to CVDs (in 000’s) by age and sex, India, 2010.

**Figure 4:**
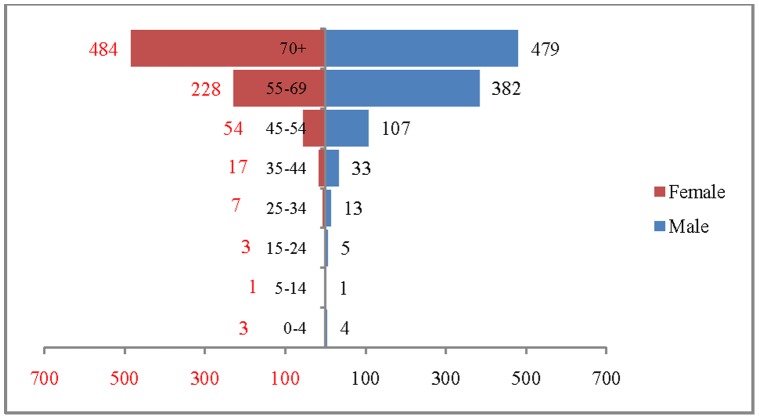
Estimated number of deaths due to CVDs (in 000’s) by age and sex, India, 2016.

**Figure 5:**
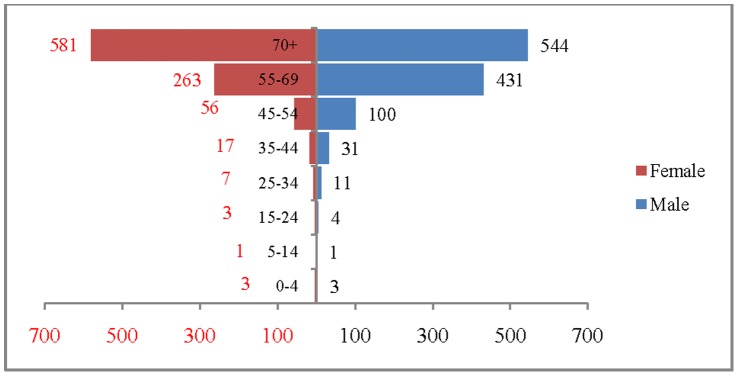
Estimated number of deaths due to CVDs (in 000’s) by age and sex, India, 2021.

The age and sex pattern of CVD deaths for 2004, 2010, 2016 and 2021 are presented in figure 2, figure 3, figure 4 and figure 5 respectively. The total number of CVD deaths is estimated at 1.4 million in 2004, 1.6 million in 2010, 1.8 million in 2016 and 2.1 million in 2026. Among males, the estimated number of CVD deaths was 0.8 million in 2004 and projected to be 1.0 million in 2016 and 1.1 million in 2021. Similarly, the estimated number of deaths due to CVDs, among females was 0.6 million in 2004, 0.8 million in 2016 and likely to be 0.9 million in 2021. The distribution of deaths by broad age group suggests that less than 1% of CVD deaths were below 25 years, 56.2% in the age group 25–69 and 41.2.% among 70 years and above, in 2004. This shows that the CVDs are affecting both working and elderly population, and the pattern remains similar till 2010. By 2021, it is projected that 45% of CVD deaths will be in the age group 25–69 and about 55% in the age group 70 years and above. The estimated number of deaths is likely to increase for all the age groups except 0–19 during the projection period. Besides the open ended age group (70+), the concentration of deaths is in the age group 55–69 during the study period. For example, the estimated number of deaths in the age group 55–69 is likely to increase from 0.49 million in 2004 to 0.59 million in 2010 and 0.69 million in 2021. The shape of the pyramid in all the three projected period continues to be inverted. The sex pattern of CVD reveals that the estimated number of deaths was higher among males than that of females.

**Figure 6:**
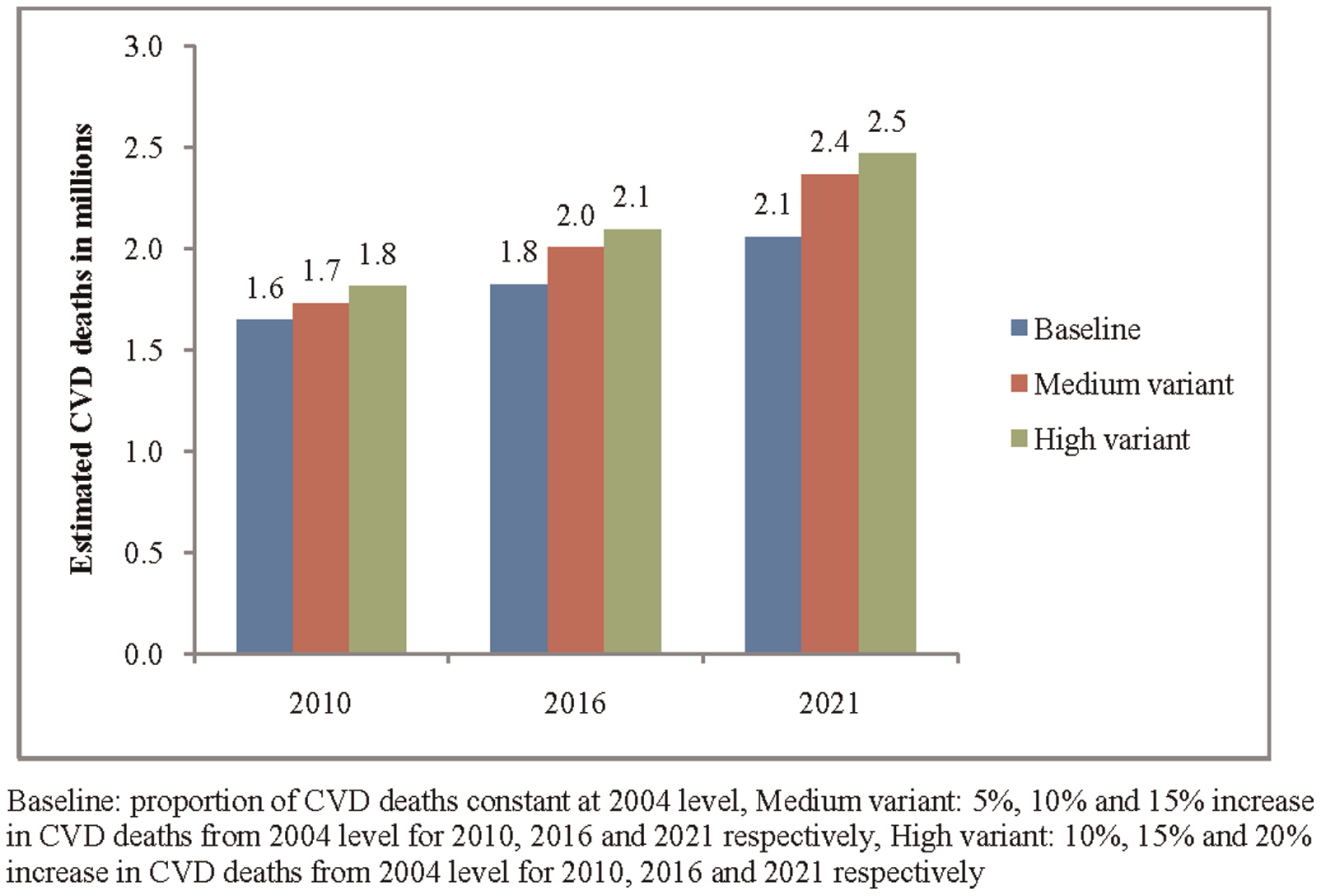
Sensitivity analyses for the estimates of CVD deaths in India, 2010–21.

Figure 6 presents the results of the sensitivity analysis for estimates of CVD deaths for 2010, 2016 and 2021. The estimated deaths in 2010 were 1.65 million under the baseline scenario, 1.73 million under medium variant and 1.81 million under high variant. The estimate varies in the range of 1.82–2.09 million for 2016 and 2.06–2.47 million for 2021. The detailed age-sex specific estimates of the sensitivity analyses are presented in Table S2.

#### Estimated hospitalisation due to cardiovascular diseases in India, 2004–21

**Figure 7:**
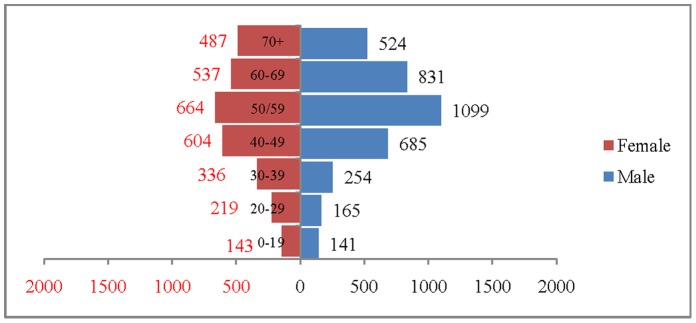
Estimated number of hospitalisation due to CVDs (in 000’s) by age and sex, India, 2004.

**Figure 8:**
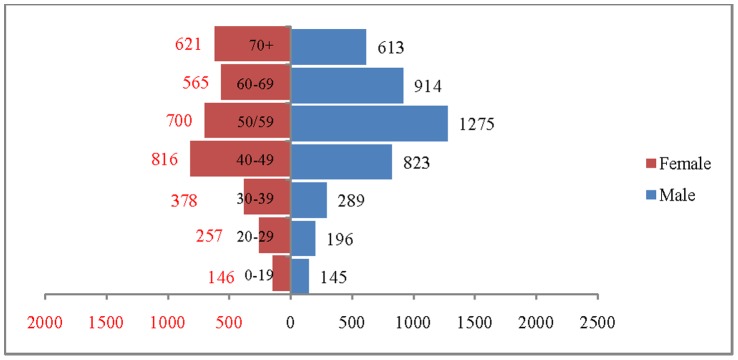
Estimated number of hospitalisation due to CVDs (in 000’s) by age and sex, India.

**Figure 9:**
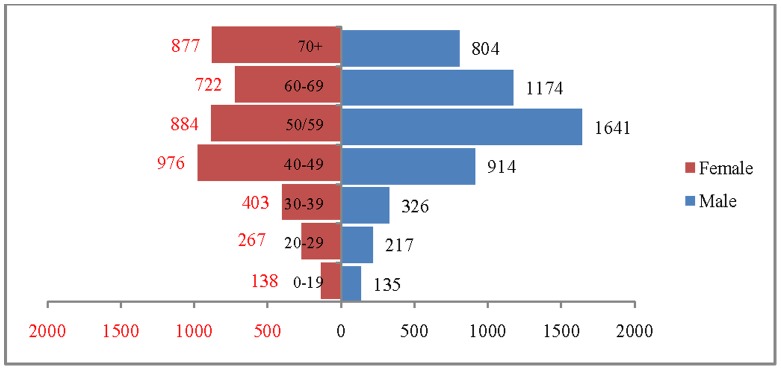
Estimated number of hospitalisation due to CVDs (in 000’s) by age and sex, India, 2016.

**Figure 10:**
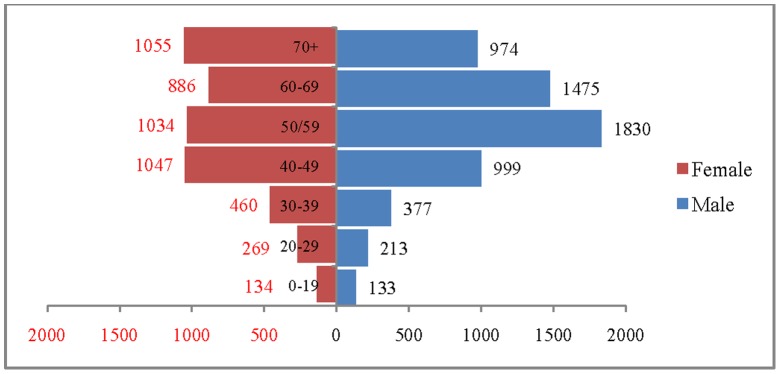
Estimated number of hospitalisation due to CVDs (in 000’s) by age and sex, India, 2021.

The age and sex pattern of hospitalisation for 2004, 2010, 2016 and 2021 are presented in Figure 7, Figure 8. Figure 9 and Figure 10 respectively. The number of hospitalisations due to CVDs was estimated at 6.6 million in 2004, 7.7 million in 2010 and 10.9 million by 2021. Among males, the estimated number of hospitalisation was 3.7 million in 2004 and projected to be 4.3 million in 2010, 5.2 million in 2016 and 6 million in 2021. Similarly, among females the estimated number of hospitalisation due to CVDs was 3 million in 2004, 3.5 million in 2010, 4.3 million in 2016 and likely to be 4.9 million in 2021. The distribution of hospitalisation in 2004 indicates that about 8% of hospitalisation was among less than 25 years, 76% in the age group 25–69 and 16% among 70 years and above. By 2021, it is projected that 5% of hospitalisation will be among those less than 25 years, 74% in the age group 25–69 and 21% in 70 years and above. The shape of the pyramid will broaden over time indicating that the estimated number of hospitalisation due to CVDs is likely to increase across all age groups. For example, the estimated number of hospitalisation in the age group 50–59 is likely to increase from 1.7 million in 2004 to 2.8 million in 2021. Of note, more than 50% of the hospitalisation due to CVDs are concentrated in the prime working age group (25–59) and will hold true for the projected years (3.7 million in 2004, 4.1 million in 2010 and 5.6 million in 2021). The estimated number of hospitalisation due to CVDs was higher among males compared to females during the projected period. For example in 2004, 3.7 million males were hospitalised compared to 3.0 million females and the differences will continue over time. On comparing the deaths and hospitalisation by age group for 2021, we found that while the CVDs deaths will be higher in older ages (70+), the hospitalisation will be higher in the working age group.

#### Estimated cost of hospitalisation for cardiovascular diseases in India, 2004–21

**Figure 11:**
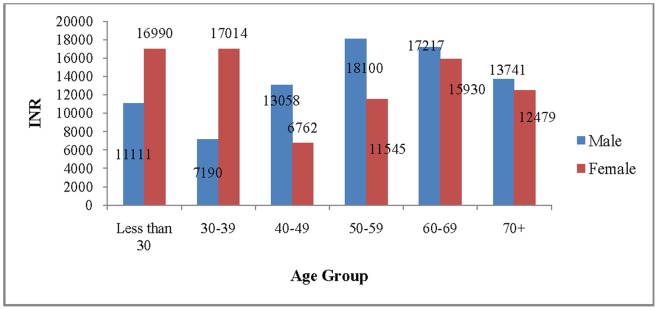
Mean cost of hospitalisation for CVDs by age and sex, India, 2004.

Figure 11 presents the mean cost of hospitalisation for CVDs by broad age group and sex. In 2004, the mean cost of hospitalisation for CVDs was Rs 14, 975/− for males and Rs 12, 276/− for females, while the median cost was Rs 4870/− for males and Rs 3500/− for females. There is no specific age pattern in cost of hospitalisation for CVDs. Among males, the mean cost of hospitalisation for CVDs was highest in the age group 50–59 and lowest in the age group 30–39. Similarly, among females, the mean cost of hospitalisation was highest in the age group 30–39 and lowest in the age group 40–49. Based on the mean cost, we have presented the macro estimates on cost of hospitalisation for CVDs in India for 2004 (Table 2). We estimated that the total cost of hospitalisation for CVDs was Rs 93.8/− billion (2004 prices) accounting for 0.42% of GDP in 2004. The estimated cost was highest in the age group 50–59. Under the constant assumption of rate of hospitalisation and cost of hospitalisation (2004 prices), the estimated cost of hospitalisation for CVDs in 2010 was Rs 107.2/− billion and likely to be Rs 131.8/− billion in 2016 and Rs 152.1/− billion in 2021 at 2004 prices. The macro estimates on cost of hospitalisation for CVDs show that about one-third of the total cost was in the age group 50–59 during all the time points.

**Table 2:** Cost of hospitalisation for cardiovascular diseases, India, 2010–21 (at 2004 prices).

	Estimated cost of hospitalisation in Indian rupees (in billions)											
Age group	2004			2010			2016			2021		
	Male	Female	Total	Male	Female	Total	Male	Female	Total	Male	Female	Total
0–19	1.3	3.9	5.2	1.3	3.9	5.2	1.2	3.7	5.0	1.2	3.6	4.8
20–29	2.1	2.2	4.4	2.5	2.6	5.2	2.8	2.7	5.5	2.8	2.8	5.5
30–39	1.8	5.7	7.6	2.1	6.4	8.5	2.3	6.9	9.2	2.7	7.8	10.5
40–49	8.9	4.1	13.0	10.7	5.5	16.3	11.9	6.6	18.5	13.0	7.1	20.1
50/59	19.9	7.7	27.6	23.1	8.1	31.2	29.7	10.2	39.9	33.1	11.9	45.1
60–69	14.3	8.6	22.9	15.7	9.0	24.7	20.2	11.5	31.7	25.4	14.1	39.5
70+	7.2	6.1	13.3	8.4	7.7	16.2	11.1	10.9	22.0	13.4	13.2	26.5
All	55.6	38.2	93.8	63.9	43.3	107.2	79.3	52.6	131.8	91.6	60.5	152.1

### Section II

#### Socio-economic differentials in cost of hospitalisation for cardiovascular diseases in india, 1995–2004

Table 3 describes the socioeconomic and demographic differentials in hospitalisation and mean cost of hospitalisation for CVDs in 1995–96 and 2004. The cost includes expenditure on doctor’s fee, medicines, diagnostic tests, bed charges, attendant charges and other non medical costs for each episode of hospitalisation. The cost of hospitalisation for CVDs for 1995–96 is adjusted to 2004 prices to nullify the price effect. During 1995–2004, the proportion of hospitalisation due to CVDs has increased significantly across all the socio-economic and demographic sub-groups. During 1995–2004, the proportion of hospitalisation due to CVDs had increased from 9.3% to 12.2% in the age group 40–59 and from 9.1% to 14.1% in the age group 60 years and above. With respect to educational attainment, we found that the increase is more pronounced among those who had educational attainment up to higher secondary and above, followed by those had education up to secondary level. Similarly, the proportion of hospitalisation due to CVDs has increased across MPCE quintiles, and the increase was significantly higher in the richest quintile. The increase in proportion of hospitalisation due to CVDs was higher in private health centres compared to public centres. The proportion of hospitalisation due to CVDs was highest among regular employee **(**those worked on regular salary/wage) followed by self employed and relatively low among low paid workers. The differentials in hospitalisations due to CVDs by geographical regions suggest that it was highest in the southern region indicating a possible association of demographic transition and the epidemiological transition.

**Table 3:** Socio-economic differentials in hospitalisation and mean cost of hospitalisation for cardiovascular diseases, India, 1995–2004.

Socio-economic and demographic variables	Proportion of hospitalisation due to CVDs			Mean cost of hospitalisation for CVDs at 2004 prices		Percentage change in mean cost
	1995–96	2004	Z statistic	1995–96	2004	
**Age**						
Less than 25 years	1.9	1.8	0.5	7851	15430	49
25–39 years	2.6	3.8	−3.8	9547	12666	25
40–59 years	9.3	12.2	−5.7	15502	13077	−19
60 years and above	9.1	14.1	−7.4	9050	15192	40
**Place of residence**						
Rural	4.6	5.9	−5.6	9392	10746	13
Urban	6.4	10.5	−9.7	16074	17923	10
**Sex**						
Male	5.3	7.5	−7.6	12441	14984	17
Female	5.0	7.1	−6.9	11645	12767	9
**Marital status**						
Never married	2.2	1.5	3.4	8683	17143	49
Currently married	5.9	9.5	−12.3	14129	14425	2
Widowed/divorced/separated	9.8	11.4	−1.9	6236	10606	41
**Education**						
Illiterate	4.5	6.0	−5.1	5175	7699	33
Primary	5.2	7.3	−5.4	8972	12189	26
Secondary	6.3	8.5	−4.6	17449	14205	−23
Higher Secondary and above	6.0	10.6	−5.4	36711	34401	−7
**Work status**						
Self employed	6.7	9.6	−5.0	9416	14816	36
Regular employee	6.5	12.3	−6.2	23131	13470	−72
Low paid workers	3.9	8.7	−8.2	5008	10606	53
Not working	4.7	5.7	−3.8	12562	15514	19
**Caste**						
SC/ST	4.0	5.3	−3.5	6882	11030	38
Others	5.6	7.9	−9.4	13257	14566	9
**MPCE Quintiles**						
Poorest	2.7	4.8	−3.5	1335	4345	69
Poor	3.6	5.9	−4.3	4230	5865	28
Middle	3.3	5.6	−5.2	3641	11582	69
Rich	6.1	8.0	−4.0	4737	12517	62
Richest	6.1	9.0	−8.3	18372	18957	3
**Type of CVD**						
Heart disease	3.2	5.0	−10.4	16221	17777	9
Hypertension	2.0	2.3	−2.8	5244	5666	7
**Type of hospital**						
Public	4.8	6.3	−5.3	6548	7430	12
Private	5.8	8.0	−7.6	16275	17411	7
**Region**						
North	7.4	7.1	0.5	9352	17223	46
East and NE	3.2	5.1	−4.6	11215	16773	33
West	5.0	8.3	−6.8	12996	12274	−6
South	6.1	8.8	−7.4	12161	12618	4
Central	3.6	5.3	−3.7	15404	15887	3
All	5.2	7.3	−10.1	12080	13963	13

Source: NSSO 52^nd^ and 60^th^ round.

The mean cost of hospitalisation for CVDs varies largely across socio-economic and demographic sub-groups. With respect to economic groups, the cost of hospitalisation for CVDs among those belonging to the richest MPCE quintile, in 2004, was four times higher than those in the poorest MPCE quintile, linking the ability to pay with treatment of CVDs. However, the differentials in cost of hospitalisation across MPCE quintiles have narrowed down over time. During 1995–2004, the increase in real cost of hospitalisation for CVDs was maximum for the poorest quintile (69%) compared with the richest quintile (3%). The mean cost of hospitalisation for CVDs by work status indicates a decline in cost of hospitalisation among regular employee. Like economic status, the mean cost of hospitalisation for CVDs was high for more educated. For example, in 2004, an illiterate spent Rs 7699/− on hospitalisation for CVDs, and those having education up to higher secondary and above, spent Rs 34401/−. Similarly, the mean cost of hospitalisation in a private health centre was two times more than that of public health centre. The mean cost of hospitalisation for CVDs was higher for those residing in urban areas than that of rural areas. During 2004, the mean cost of hospitalisation was highest in the northern region and lowest in the western region.

#### Predictors of cost of hospitalisation for cardiovascular diseases in India

Table 4 presents the results of the OLS regression where the dependent variable is the logarithm of cost of hospitalisation for CVDs. The independent variables are age, sex, place of residence, marital status, caste, educational attainment, work status, monthly per capita consumption expenditure (MPCE), type of hospital and states based on fertility levels. The states were classified on the basis of crude birth rate (CBR) of 19 or less, 19.1–23 and more than 23. The continuous variables age and MPCE were log transformed. The regression coefficients are in expected direction and support the bi-variate analyses. The significant predictors in the model are MPCE, educational attainment, type of hospital, level of fertility in the states, place of residence and occupation. With respect to MPCE, the coefficient was positive and statistically significant indicating that economically better off households demand for better health services. The cost of hospitalisation in private health centres was 89% higher than that of public health centres. As compared to low fertility states, the cost of hospitalisation for CVDs was 33% higher in moderate fertility states and 55% higher in high fertility states. This may be attributed to the availability of better and low cost curative services for non communicable diseases in low fertility states.

**Table 4:** Results from log transformed ordinary least square regression on cost of hospitalization in India.

	β Coefficients	T statistic	Confidence interval
**Log (age)**	0.01	0.17	(−0.14–0.17)
**Place of residence**			
Rural			
Urban	−0.14	−2.22	(−0.33−0.05)
**Sex**			
Male			
Female	−0.23	−1.46	(−0.44– −0.03)
**Marital status**			
Currently married			
Others	0.18	1.69	(−0.03–0.39)
**Education**			
Illiterate			
Primary	0.27	2.40	(0.05–0.49)
Secondary	0.37	3.05	(0.13–0.61)
Higher Secondary and above	0.51	2.88	(0.16–0.85)
**Work status**			
Self employed			
Regular employee	−0.37	−2.12	(−0.72–−0.03)
Low paid workers	−0.05	−0.37	(−0.34–0.23)
Not working	0.10	0.74	(−0.16–0.35)
**Caste**			
SC/ST			
Others	0.25	1.78	(−0.03−0.52)
**Log (MPCE)**	0.40	4.22	(0.22–0.59)
**Type of hospital**			
Public			
Private	0.89	9.17	(0.70–1.07)
**States**			
Low fertility			
Moderate fertility	0.33	3.17	(0.13–0.54)
High fertility	0.55	5.39	(0.35–0.75)
**Constant**	3.54	5.77	(2.34–4.74)

Source: NSSO 60^th^ round.

## Discussion

India, like many transitional economies, is undergoing rapid demographic and epidemiological transition. The demographic transition, characterised by an increase in longevity and reduction in fertility, has begun to alter the age-sex structure of the population. The age-sex structural transition, coupled with epidemiological transition has seen a rising trend in CVD burden in India. Though the CVDs are the leading cause of mortality and one of the major causes of hospitalisation in India, little is known about the magnitude of deaths, hospitalisation and associated cost of treatment. Most of the writings on CVDs in India are based on small scale un-representative studies and limited to analysing the risk factors [29], [50], [51]. Therefore, the first logical step in this direction is to provide a numerical estimate of mortality, hospitalisation and cost of hospitalisation due to CVDs. This is because many of the CVD deaths are premature and avoidable. However, there is no single comprehensive data source in India that provides information on the deaths, disease pattern and the cost of treatment for CVDs. The aim of this paper is to project the deaths, hospitalisation and cost of hospitalisation for cardiovascular diseases in India.

The following are the salient findings of our study. First, our results indicate a sharp increase in deaths and hospitalisation due to CVDs across age groups in coming years. During 2004–21, the estimated deaths and hospitalisation due to CVDs is likely to increase from 1.4–2.1 million and 6.7–10.8 million respectively. It is projected that one-third of the deaths and half of the hospitalisation due to CVDs will be in the working age group (25–59) that are premature and avoidable. The increase in mortality and hospitalisation is largely attributed to the rise in the risk factors of CVDs and rapid age-structural transition. Majority of hospitalisation due to CVDs will be in the working age group indicating that a large young population is at risk. If timely and proper intervention is provided, it may reduce the premature mortality and burden of disease. Our estimates on CVD deaths for 2010 are lower than the WHO estimates for 2008 because of the methodological differences [52]. Second, both hospitalisation and mortality were higher among males than females across age group, over time. This may be due to the fact that the risk factors like smoking, alcohol consumption and work related stress are higher among males than females [53], [54]. Third, the increase in the cost of hospitalisation was high among illiterate, poor and those in low paid jobs. Adjusting for confounders, the cost of hospitalisation was high in private health centres, rural areas, and in high fertility states. Though studies suggest that the treatment of CVDs requires specialised medical care and highly expensive [18], [35], the recent Lancet Series argue that low cost and effective interventions do exist and stressed the need of political commitment for reduction of NCDs [15], [16], [55], [56]. The cost of treatment was higher in the northern region as compared to the western region. This is possibly due to limited availability of health centres in selected urban pockets and varying fees in the private health centres for treatment of CVDs in the northern region. Fourth, the proportion of hospitalisation due to CVDs has increased over time, and we found a positive socio-economic gradient for CVD hospitalisation. The findings are consistent with many studies in the developing countries [34], [57]–[61].

Based on the findings, we suggest an increase in the budgetary allocation for non-communicable diseases, in order to expand and modernise the existing infrastructure for treatment of CVDs. Though the Government of India has increased the health spending substantially, little has been earmarked for non-communicable disease. Second, we recommend formulating comprehensive strategies for prevention and control of CVDs. These include strategies on the reduction of risk factors, revamping the public health centres, accreditation of private health centres to provide cost-effective treatment and care, increasing awareness on healthy life style through mass media, free health check up and insurance coverage. Though the National Programme for Prevention and Control of Cancer, Diabetes, Cardiovascular Diseases and Stroke (NPCDCS) was launched in 2010 with the primary objective of reducing the risk factors of NCDs in the community, the coverage of the programme is limited [62]. Third, we recommend launching a population based study to bridge the data gap and generate evidence on the prevalence, socio-economic differentials and risk factors of non-communicable diseases. Though a number of population based representative studies have guided us in arriving at evidence based policies to improve the maternal and child health, there are no such studies on non-communicable diseases. Such evidences will be helpful to plan and develop a comprehensive strategy for the prevention, control and treatment of CVDs and other non communicable diseases.

We outline the following limitations of our study. First, the cross sectional data are compiled from the two main sources, namely SSD 2001–03 and the NSS and integrated with the population estimates by age and sex. While the NSS provides the unit data on morbidity that facilitate detailed analyses, the SSD provides only aggregate data, and so mortality differentials were not analysed. Second, the sample size was limited to carry out disaggregated analyses by disease and regions. Third, though the NSS provides the hospitalisation and cost of treatment, there are limited variables on the risk factors of CVDs. Also, the estimates on cost are limited to direct cost. Despite these limitations, the strength of this paper is the use of multiple data source and a simplified methodology to arrive at the macro estimates of deaths, hospitalization and cost of hospitalisation for CVDs. Our estimates for 2004 are actual and that for the successive years are projected under constancy in the age pattern of CVD deaths and hospitalisation. Given the current state of epidemiological transition and rise in the risk factors, the CVD deaths and hospitalisation is likely to increase in coming years. Though our estimates are minimum, they provide an insight into the future magnitude of the problem and underscore the need to develop strategies to save human life and medical cost.

## Supporting Information

Table S1 **Proportion of CVD hospitalization, mean cost of hospitalization and 95% Confidence interval, India, 2004.**
(DOCX)

Table S2 **Sensitivity analyses on estimates of CVD deaths by age and sex, India, 2010–21.**
(DOCX)
