# Supplementary material for: Age and Sex Pattern of Cardiovascular Mortality, Hospitalisation and Associated Cost in India
Source: PLoS One. 2013 May 7;8(5):e62134. doi: 10.1371/journal.pone.0062134 (PMC3646767; doi:10.1371/journal.pone.0062134)
Supplement: Table S1 — Proportion of CVD hospitalization, mean cost of hospitalization and 95% Confidence interval, India, 2004. (DOCX) [file pone.0062134.s001.docx]

Table S1: Proportion of CVD hospitalization, mean cost of hospitalization and 95% Confidence interval, India, 2004

|  | Hospitalization | | Mean Cost of hospitalization | |
| --- | --- | --- | --- | --- |
| Age group | Mean | CI (95%) | Mean | CI (95%) |
| 0-19 | 0.013 | (0.011- 0.016) | 18235 | (4216-32254) |
| 20-29 | 0.028 | (0.023-0.032) | 11357 | (6422- 16293) |
| 30-39 | 0.046 | (0.040-0.052) | 12784 | (6963 -18605) |
| 40-49 | 0.103 | (0.094-0.111) | 9939 | (7810 -12068) |
| 50/59 | 0.144 | (0.134-0.155) | 15628 | (12407 -18850) |
| 60-69 | 0.146 | (0.134-0.159) | 16675 | (11907 -21442) |
| 70+ | 0.134 | (0.120-0.148) | 13129 | (10163 -16095) |
