# Supplementary material for: Age and Sex Pattern of Cardiovascular Mortality, Hospitalisation and Associated Cost in India
Source: PLoS One. 2013 May 7;8(5):e62134. doi: 10.1371/journal.pone.0062134 (PMC3646767; doi:10.1371/journal.pone.0062134)
Supplement: Table S2 — Sensitivity analyses on estimates of CVD deaths by age and sex, India, 2010–21. (DOCX) [file pone.0062134.s002.docx]

Table S2: Sensitivity analyses on estimates of CVD deaths by age and sex, India, 2010-21

| **Person** | 2010 | | | 2016 | | | 2021 | | |
| --- | --- | --- | --- | --- | --- | --- | --- | --- | --- |
|  | Baseline | Medium variant | High variant | Baseline | Medium variant | High variant | Baseline | Medium variant | High variant |
| 0-4 | 6315 | 6631 | 6946 | 6774 | 7451 | 7790 | 5686 | 6539 | 6823 |
| 5-14 | 3570 | 3748 | 3927 | 2180 | 2398 | 2507 | 1810 | 2081 | 2172 |
| 15-24 | 23974 | 25172 | 26371 | 8436 | 9279 | 9701 | 6786 | 7804 | 8143 |
| 25-34 | 46408 | 48728 | 51048 | 19779 | 21757 | 22746 | 18096 | 20810 | 21715 |
| 35-44 | 93026 | 97678 | 102329 | 50026 | 55028 | 57530 | 47800 | 54969 | 57359 |
| 45-54 | 188069 | 197472 | 206876 | 160935 | 177029 | 185075 | 156306 | 179752 | 187567 |
| 55-69 | 595592 | 625372 | 655151 | 610343 | 671377 | 701894 | 693881 | 797963 | 832657 |
| 70+ | 690741 | 725279 | 759816 | 963594 | 1059953 | 1108133 | 1125804 | 1294675 | 1350965 |
| Total | 1647695 | 1730080 | 1812464 | 1822065 | 2004271 | 2095375 | 2056168 | 2364593 | 2467401 |
| **Male** |  |  |  |  |  |  |  |  |  |
| 0-4 | 3158 | 3316 | 3474 | 3694 | 4063 | 4248 | 2953 | 3396 | 3543 |
| 5-14 | 1633 | 1715 | 1797 | 1097 | 1206 | 1261 | 861 | 990 | 1033 |
| 15-24 | 12191 | 12801 | 13410 | 4992 | 5491 | 5740 | 3897 | 4482 | 4676 |
| 25-34 | 29449 | 30922 | 32394 | 12952 | 14247 | 14894 | 11392 | 13100 | 13670 |
| 35-44 | 66647 | 69979 | 73311 | 33013 | 36314 | 37964 | 31135 | 35805 | 37362 |
| 45-54 | 131202 | 137762 | 144322 | 106571 | 117228 | 122557 | 99935 | 114925 | 119922 |
| 55-69 | 367749 | 386136 | 404523 | 382433 | 420676 | 439798 | 430695 | 495300 | 516834 |
| 70+ | 369150 | 387608 | 406065 | 479429 | 527372 | 551343 | 544431 | 626096 | 653318 |
| Total | 981179 | 1030238 | 1079297 | 1024179 | 1126597 | 1177806 | 1125299 | 1294094 | 1350359 |
| **Female** |  |  |  |  |  |  |  |  |  |
| 0-4 | 3157 | 3315 | 3473 | 3080 | 3388 | 3542 | 2733 | 3143 | 3280 |
| 5-14 | 1937 | 2033 | 2130 | 1083 | 1191 | 1245 | 949 | 1091 | 1138 |
| 15-24 | 11783 | 12372 | 12961 | 3444 | 3788 | 3961 | 2889 | 3323 | 3467 |
| 25-34 | 16958 | 17806 | 18654 | 6827 | 7510 | 7851 | 6704 | 7710 | 8045 |
| 35-44 | 26380 | 27699 | 29018 | 17013 | 18715 | 19565 | 16665 | 19164 | 19998 |
| 45-54 | 56867 | 59710 | 62553 | 54364 | 59800 | 62518 | 56371 | 64826 | 67645 |
| 55-69 | 227843 | 239236 | 250628 | 227910 | 250701 | 262096 | 263185 | 302663 | 315822 |
| 70+ | 321591 | 337671 | 353750 | 484165 | 532581 | 556790 | 581373 | 668579 | 697647 |
| Total | 666515 | 699841 | 733167 | 797886 | 877674 | 917569 | 930869 | 1070499 | 1117043 |
